# Benefits and obstacles of interdisciplinary research: Insights from members of the Young Academy at the Heidelberg Academy of Sciences and Humanities

**DOI:** 10.1016/j.isci.2023.108508

**Published:** 2023-12-12

**Authors:** Christian A. Mahringer, Franziska Baessler, Martin F. Gerchen, Christoph Haack, Katharina Jacob, Simone Mayer

**Affiliations:** 1 WIN-Kolleg, Heidelberg Academy of Sciences and Humanities, Karlstraße 4, Heidelberg, Baden-Wuerttemberg 69117, Germany; 2 University of Stuttgart School of Management, Keplerstraße 17, Stuttgart, Baden-Wuerttemberg 70174, Germany; 3 Department of General Internal Medicine and Psychosomatics, Center for Psychosocial Medicine, Heidelberg University Hospital, Im Neuenheimer Feld 410, Heidelberg, Baden-Wuerttemberg 69120, Germany; 4 Central Institute of Mental Health, Medical Faculty Mannheim, University of Heidelberg, J 5, Mannheim, Baden-Wuerttemberg 68159, Germany; 5 CRC 923 “Threatened Order”, University of Tübingen, Doblerstraße 21, Tübingen, Baden-Wuerttemberg 72074, Germany; 6 Department of German Studies, University of Heidelberg, Hauptstraße 207-209, Heidelberg, Baden-Wuerttemberg 69117, Germany; 7 Hertie Institute for Clinical Brain Research, University of Tübingen, Otfried-Müller-Str. 25, Tübingen, Baden-Wuerttemberg 72076, Germany

**Figure:**
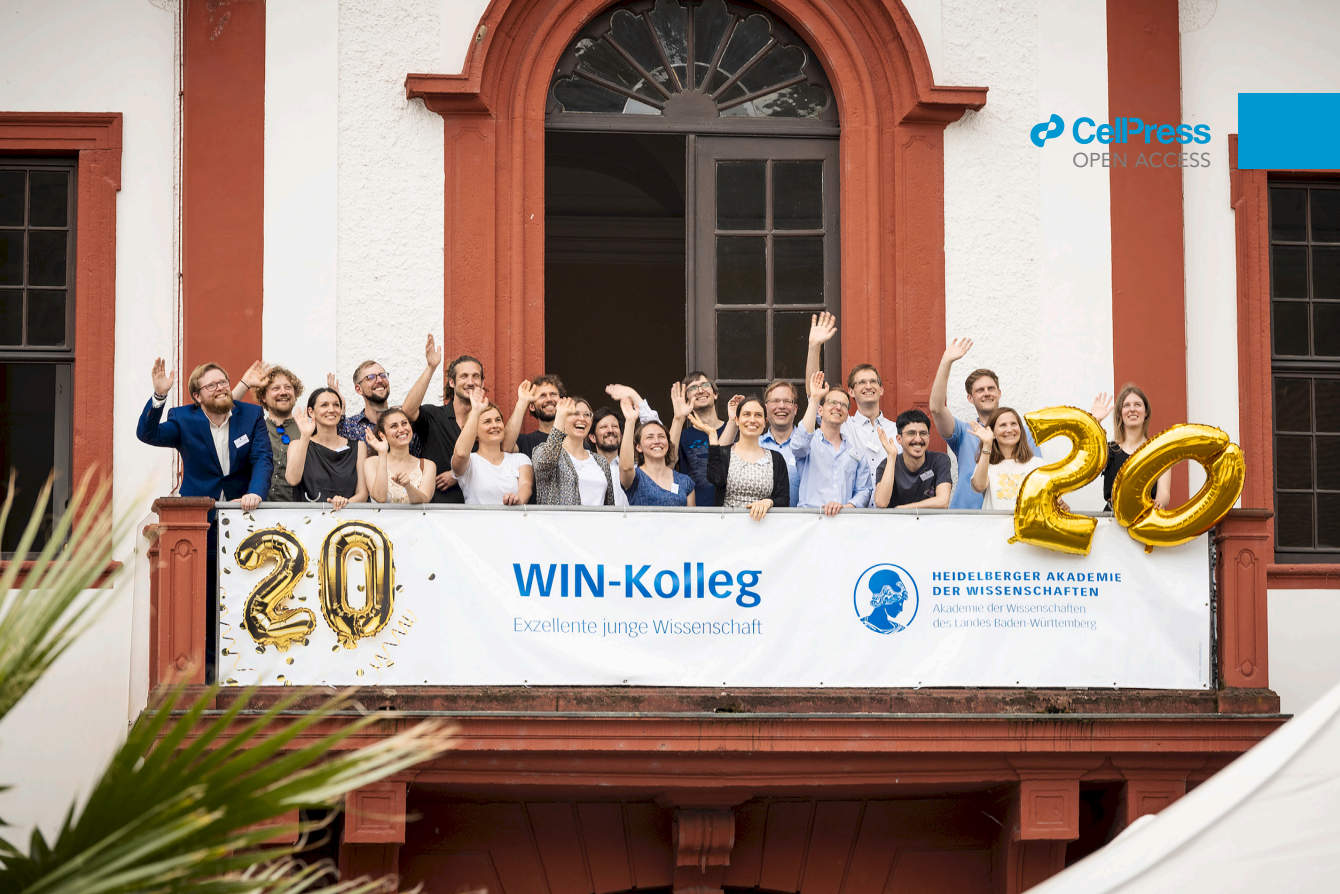
Above image: Members of the Young Academy at the Heidelberg Academy of Sciences and Humanities Photo: HAdW/Tobias Schwerdt.

The opportunity to see and work beyond the overarching boundaries of specific disciplines has provided us with a path of immense personal development and discovery.

Interdisciplinary projects offer the chance to reflect on own scientific procedures, further develop the own disciplinary understanding, and become aware of the borders of the own discipline.

It is our experience that the encounter with other scholars and subjects does indeed inevitably change one’s own view of the own discipline.

In the narratives of the others, the world of the other, the (subject-) cultural, social, situational realities become visible, and in this way, we can communicate empathically, develop understanding in interdisciplinary conversation, and deal with the missing knowledge of our own subject and the other subjects.

As we delved into our interdisciplinary project, we witnessed the emergence of a “third realm,” an intellectual environment where we scrutinized a research topic that we had not initially planned for at the beginning of the project.

Engagement in interdisciplinary research may be a question of personality and an opportunity to define oneself as a researcher in a broader societal context.

Interdisciplinarity is often considered a key element of successful research projects.^1^ Funding organizations, for instance, promote interdisciplinarity by making it a key criterion for the selection of projects. Moreover, interdisciplinary work may help to overcome demanding situations like the COVID-19 pandemic.^2^ While it is clear that interdisciplinarity is a target of policymakers and funding organizations, it is not entirely clear what the benefits of such research projects are and how they can be successfully conducted.^3^^,^^4^^,^^5^

In this Backstory, we aim to shed more light on these aspects. We offer a reflection of our own experiences with interdisciplinary research in the Young Academy of the Heidelberg Academy of Sciences and Humanities, a scholarly society and non-university research organization in Baden-Wuerttemberg, Germany. The authors are fellows of the 7^th^ program (topic: “Collective decision-making”) and the 8^th^ program (topic: “Stability and instability”) of the WIN-Kolleg, which is an essential part of the Young Academy: in regular intervals, the Heidelberg Academy of Sciences and Humanities funds promising scholars (i.e., postdocs, research group leaders) who intend to conduct research projects over a period of three to five years within this program. The WIN-Kolleg is a prototypical example of the role interdisciplinarity plays for funding organizations. Only project teams that involve multiple disciplines are eligible for this funding, and developing scientific insights in an interdisciplinary way is a central goal of each project.

Over the last years, each one of us has collaborated intensively with scholars from other disciplines within and beyond our research projects. The question that we would like to shed light on in this Backstory is: “What are the benefits and obstacles of interdisciplinary research projects?” We not only intend to highlight the advantages of such research but also take a critical perspective. The value of our reflection lies in providing real-life experiences of interdisciplinary research, which may enable policymakers and funding organizations to shape and improve their practices. Moreover, we believe that our article may help other scholars to strengthen their interdisciplinary research endeavors. The article is structured as a collection of different opinions: each author provides his or her stance on the notion of interdisciplinary research, based on the experiences gathered in our projects. In conjunction, these contributions can serve as a resource for scientists and policymakers alike, providing guidance and support for the effective implementation and facilitation of interdisciplinary research endeavors.

### Personal development: Why interdisciplinary teamwork matters

#### Franziska Baessler, Medicine

In 2015, Nature announced in one of its special issues: “Scientists must work together to save the world”.^6^ The edition was aptly named “Why interdisciplinary research matters?”, and discussed the measures on how to make interdisciplinary research work. Despite immense challenges, the interdisciplinary model of scientific research has proliferated in the past two decades, countering the “specialization model” that had dominated science since WWII.^7^ While the interest in multidisciplinary and interdisciplinary research has expanded worldwide, researchers are still primarily individuals from single disciplinary backgrounds. Not only does this lead to personal and professional challenges but does it also open potential avenues of personal growth and professional collaboration.

In the experience we gained in our teamwork, interdisciplinary research has challenged our contemporary beliefs about bounded research disciplines since our project lies at the crossroads of theoretical physics, psychology and medicine; starkly different disciplines with rare points of overlap. The opportunity to see and work beyond the overarching boundaries of specific disciplines has provided us with a path of immense personal development and discovery. After more than two years of working together, we have experienced ideological changes of perspectives and broadening of horizons in the scientific thought process. This not only gives researchers the ability to see beyond their discipline and be respectful of the other perspective, but also allows them to self-reflect on the gaps in scientific knowledge and methodologies within their disciplines.

Working in interdisciplinary teams creates a learning environment conducive to open exchanges and interprofessional teamwork, which in turn can help researchers to respect, appreciate and learn from their colleagues in other disciplines and counter the existing prejudices. Since they face similar challenges in their research work, interdisciplinary scientists can use these opportunities to enrich their experiences and gain a holistic understanding of common problems and be more respectful and sympathetic toward each other. Consequently, harmony, satisfaction and personality development become a part of scientific training and can help researchers to be more resilient toward challenging situations that otherwise lead to frustration and burnout.

### Interdisciplinary work sharpens the own disciplinary research profile

#### Martin F. Gerchen, Biological psychology

Besides providing individual learning experiences and broadening the content of the conducted research, in our experience, being involved in interdisciplinary research also has a substantial influence on the own disciplinary research. In addition to reflecting the own scientific language, learning languages of other disciplines, and developing a common language, similar processes are also occurring with respect to the basic ideas, concepts and assumptions that are characteristic for a specific scientific discipline. Conducting interdisciplinary research offers the opportunity to become aware of the underlying narratives and basic scientific concepts that are commonly used in a discipline by comparing them with those of other disciplines. Obviously, such awareness can also arise from reflection within a discipline, and should be part of scientific behavior in general, but it seems much harder to identify one’s own scientific neglects and peculiarities without external reference. In interdisciplinary projects, discussions about the differential understanding of science and the blind spots of the perspectives arise almost inevitably. If these debates are held with mutual sympathy in a benevolent way, each involved discipline can benefit tremendously by reflecting on the limits and strengths of its own procedures and approaches.

In our interdisciplinary project (8^th^ program of the WIN-Kolleg, project “Heterodoxy as factors of stability”) we are bringing together the fields of philosophy, German literature science, computational neuroscience, and psychology to study beliefs in alternative medicine, pseudoscience, and conspiracy theories. Thus, we are approaching a common topic from a humanities as well as a cognitive neuroscience perspective. In our work, the natural science perspective was especially enriched by the sharp thoughts, precise definitions, and strict concepts of philosophy, and the insights that can be obtained from individual autobiographical texts. On the other hand, for the humanities it was important to see how ideas and concepts can be tested experimentally and how the results of experimental studies can influence theoretical understanding. From these experiences we have gained a deeper understanding of our own scientific fields, which will also influence our future disciplinary work.

In conclusion, from our experience, interdisciplinary projects (if they work well) offer the chance to reflect on own scientific procedures, further develop the own disciplinary understanding, and become aware of the borders of the own discipline. They are thus also a unique opportunity for sharpening the disciplinary research profiles of researchers.

### Knowledge development and learning

#### Christoph Haack, Medieval history

One of the most basic promises of interdisciplinary cooperation probably lies in the hope of mutual learning—of an advance of knowledge that consists in the experiences of other disciplines and people.^8^^,^^9^ The underlying idea here seems to be that the combination of expertise will inevitably result in an augmentation of knowledge: by a mere process of addition, but even more through some sort of cross-fertilization.

And to some extent, this idea seems to be quite justified. Intellectual enrichment and new perspectives opened by hitherto unknown methods do represent the strong common experiences of the WIN-Kolleg. At the same time, however, there are significant problems.^10^ First of all, the distance between disciplines is often so great that real methodological cooperation is hardly possible: the work of a theoretical physicist and an early medieval historian can barely be brought together.^11^ Even if, for example, both are interested in theories of “systems,” they define fundamentally different things under this term: from a medievalist’s perspective, the term “system” may open up an interestingly new view of familiar phenomena, such as political communities or networks of people, which, however, may not meet the definitional criteria of a physical “system.” If such different approaches are brought together, as happens in tax-funded interdisciplinary research projects, cooperation is often forced. A topic is set by the name of the program (for example, the 7^th^ program of the WIN-Kolleg is labeled “Collective decision-making”), and researchers must create meaning.

Still, here as always, every barrier can become an avenue: sometimes new approaches grow out of the problems that have been named. This can consist in the transfer of methods, both, from neighboring disciplines such as literary narratology and medieval history (7^th^ program of the WIN-Kolleg, project “Holy lives”), but maybe more surprisingly in the cooperation of physical science with linguistic and evolutionary biology (7^th^ program of the WIN-Kolleg, project “Modeling – decision making”). In this example, scholars from the fields of linguistics and biology adopt the methods of physics, thus expanding their range of methods, while scholars of physics explore new subject areas.

Such genuine collaborations do occur, but not necessarily without external “pressure” due to funding requirements, and they cannot be expected in all constellations. Still, on the level of a broadening and transformation of perspective, interdisciplinary cooperation is always a gain: it is our experience that the encounter with other scholars and subjects does indeed inevitably change one’s own view of the own discipline. However, this often takes the form of soft skills, the practical benefits of which are often not immediately apparent and do not necessarily come to pass.

### Specialized and linguistic cultures, levels of conversation and the relevance of empathy

#### Katharina Jacob, Linguistics

Every linguistic utterance is linked to knowledge. When we talk about our research subjects as scientists, however, this knowledge is not associated with the attribute “unquestionable” (according to the motto “I simply know that”), which represents a stark contrast to everyday communication. Knowledge in science communication is questionable and designed to be revised.

In specialized language research, the language of science is often referred to as distinct from other specialized languages (such as administrative language or similar), although this is the same as with English, German or other linguistic cultures: just as there is not “one” English language, there is neither one scientific language. Interdisciplinary scientific communication faces the challenge that the common object of research is not practiced communicatively within one subject or language culture of science, but that different phenomena internal and external to the language clash across subject boundaries.

Every conversation, even within a discipline, entails that not only one or more subjects of conversation, i.e., the contents, are discussed. The participants in the conversation always simultaneously engage in a rarely verbalized meta-conversation, in which, for example, the right to speak is negotiated, understanding is secured, social roles are defined, and much more.

In interdisciplinary conversation, these two levels are joined by three additional levels: each discipline must attract attention to the aspects that make it relevant to the conversation. In this transformation process, the form and the content of the utterance change. While the process of decoding occurs on this third level, the scientists have to explain this process of decoding meta-communicatively on the fourth level in order to counteract misunderstandings. Through the third and fourth levels, the participants in the conversation can find a common language, a specific interdisciplinary technical language. The fifth level of conversation can be compared to the second, because it is the level in which not only the concrete conversation is evaluated and controlled, but also in which the different professional culture-specific practices are negotiated and coordinated. At this level, a distinct interdisciplinary subject culture might develop. For example, traditional understandings of roles emerge from the disciplines to form a new code of conduct.

If we consider the course of an interdisciplinary conversation, then conversation levels 1, 3, and 4 ideally run sequentially. Conversation levels 2 and 5 run parallel to conversation levels 1, 3, and 4 and are rarely verbalized explicitly. It is helpful to be aware of these levels (see Table 1) to counteract misunderstandings and to work out comprehension together.

**Table 1:** Levels of conversation

Level of conversation 5	Negotiation and coordination of subject culture-specific practices (here, a distinct interdisciplinary subject culture might develop)
Level of conversation 4	Metacommunication via decoding (is often made explicit and, together with conversation level 3, can lead to specific interdisciplinary technical language)
Level of conversation 3	Decoding of the disciplines involved (transformation processes can lead to a specific interdisciplinary language)
Level of conversation 2	Meta-conversation about conversation (is often not or only selectively made explicit, and therefore runs implicitly and parallel to the conversation about the object of research)
Level of conversation 1	Conversation about the object of research

In this context, the concept of empathy could be helpful. In a sense, it is a linguistic utopia to think that we can put ourselves in the other person’s professional or linguistic culture. We cannot take the place of the other. We need language and interdisciplinary conversation that we quietly unfold on the five levels described so that we can immerse ourselves in the world of the other, as a kind of self-experience of the other (Liebert^12^ speaks of immersion in this context). In the narratives of the others, the world of the other, the (subject-)cultural, social, situational realities become visible, and in this way, we can communicate empathically, develop understanding in interdisciplinary conversation, and deal with the missing knowledge of our own subject and the other subjects.^13^

### Pursuing novel ideas in interdisciplinary projects

#### Christian A. Mahringer, Organization studies

A key reason for promoting interdisciplinary research lies in its assumed potential to foster creativity and innovation. However, the relationship between interdisciplinarity and innovation is not straightforward—it may promote innovation because it creates new connections, but it may also be associated with ambiguity and quality issues.^14^ When funding organizations advocate for interdisciplinary research, it becomes essential to ask, “What is it that science should gain from these projects?” and “What are the costs involved?” While these questions cannot be answered with certainty (as innovations are, by definition, unpredictable), posing and reflecting on them may help shift the role of interdisciplinarity from an *end* in itself to a *means* of achieving specific goals.

My experience from the WIN-Kolleg is that interdisciplinarity can indeed promote the emergence of innovative ideas. In our project, “Stabilizing and destabilizing processes of change” (8^th^ program of the WIN-Kolleg), for instance, we sought commonalities in processes of change across disciplines. As we engaged with this question, we realized that we first required a better understanding of which scientific communities deal with the notion of change. Consequently, we conducted a bibliometric analysis of the change literature across disciplines, which led us to develop a template to describe processes of change. These ideas emerged from our attempt to identify commonalities across disciplines, going beyond our initial intentions at the beginning of the project. As we delved into our interdisciplinary project, we witnessed the emergence of a “third realm,” an intellectual environment where we scrutinized a research topic that we had not initially planned for at the beginning of the project.

From the perspective of funding organizations, this implies that the emergence of novel ideas during the project life cycle should be encouraged. This issue further reflects what management scholars refer to as the relationship between structure and novelty in innovating.^15^ We experienced a high degree of openness in the Heidelberg Academy of Sciences and Humanities for pursuing our ideas, which enabled us to further enact them. Had the funding organization asked us to strictly adhere to our initial project description, we would not have been able to explore these valuable avenues. Moreover, the commitment to our project (e.g., in terms of work contracts and progress reports) motivated us to further pursue interdisciplinary ideas, even when their publication potential was unclear, and they required us to step out of our comfort zones. These issues are also linked to difficulties in assessing and determining the innovative potential of such interdisciplinary ideas, *a priori*.

### Interdisciplinary research—Crucial or detrimental to the development as a scientist?

#### Simone Mayer, Neurobiology

Since science is competitive and scientists regularly compete for limited funding and positions, the impact of any activity on career progression needs to be considered. This is especially true for junior scientists who do not have a permanent position yet. Interdisciplinary research may be regarded as a brake to career progression for several reasons. First, since interdisciplinary exchange comes with the cost of developing shared scientific knowledge (see section by C. Haack) and adopting a common language (see section by K. Jacob), the output is likely to be limited in the beginning compared to uni-disciplinary research. Second, dissemination and exploitation of research results are more difficult to achieve since means of publication in one discipline may not be relevant in the other discipline. Few interdisciplinary outlets exist with varying adoption and esteem in the different research communities. The audience of the interdisciplinary article is also often unclear, making a convincing writing and straightforward review process difficult. Similarly, hiring committees and grant evaluation panels may not know how to assess interdisciplinary research, thus, making the direct positive impact on one’s career progression more difficult to assess.

However, I also see several benefits to career progression through interdisciplinary research. First, interdisciplinarity is a requirement in many job descriptions and a feature evaluated in many funding calls. Being able to demonstrate interdisciplinarity should therefore be a benefit for applicants. Second, interdisciplinary research in small teams and larger teams, as, for example, the WIN-Kolleg we are part of, brings with it the establishment of new networks. The ties in these networks may be rather weak based on the distance of the different disciplines but may be strengthened by other commonalities. For example, in our cohort that is composed of scientists at a similar career stage, shared challenges based on this career stage in a specific location (for us the state of Baden-Wuerttemberg) can help to overcome disciplinary distance. Finally, knowledge of other research fields and players in these fields may help exploiting research findings in one’s own discipline more broadly and swiftly in the future.

Therefore, I propose that the long-term benefits of interdisciplinary exchange, while they can only be determined in retrospect, are likely to outweigh downsides. Importantly, I believe that the relevance of research to societal problems increases through interdisciplinary exchange and thus ultimately is a significant contributor to public acceptance and return on public investment. Finally, engagement in interdisciplinary research may be a question of personality and an opportunity to define oneself as a researcher in a broader societal context.

### Conclusion

In conclusion, we assert that interdisciplinary research contributes to the development of valuable skills and promotes meaningful dialogues, ultimately placing research within a broader societal context. This enhances the significance of research, strengthens engagement in dissemination efforts, and bolsters the societal rationale for research. Consequently, we propose that interdisciplinary research serves as a catalyst for connecting science and society more closely.
